# Effect of myeloid differentiation primary response gene 88 on expression profiles of genes during the development and progression of *Helicobacter*-induced gastric cancer

**DOI:** 10.1186/s12885-017-3114-y

**Published:** 2017-02-15

**Authors:** Ivonne Lozano-Pope, Arnika Sharma, Michael Matthias, Kelly S. Doran, Marygorret Obonyo

**Affiliations:** 1 Department of Medicine, University of California, La Jolla, CA USA; 2 Department of Biology, San Diego State University, San Diego, CA USA

**Keywords:** *Helicobacter*, MyD88, Gene regulation, Gastric cancer, Microarray

## Abstract

**Background:**

Gastric cancer is one of the most common and lethal type of cancer worldwide. Infection with *Helicobacter pylori* (*H. pylori*) is recognized as the major cause of gastric cancer. However, it remains unclear the mechanism by which *Helicobacter* infection leads to gastric cancer. Furthermore, the underlying molecular events involved during the progression of *Helicobacter* infection to gastric malignancy are not well understood. In previous studies, we demonstrated that that *H. felis*-infected *Myd88* ^−/−^ mice exhibited dramatic pathology and an accelerated progression to gastric dysplasia; however, the MyD88 downstream gene targets responsible for this pathology have not been described. This study was designed to identify MyD88-dependent genes involved in the progression towards gastric cancer during the course of *Helicobacter* infection.

**Methods:**

Wild type (WT) and *Myd88* deficient mice (*Myd88* ^−/−^) were infected with *H. felis* for 25 and 47 weeks and global transcriptome analysis performed on gastric tissue using MouseWG-6 v2 expression BeadChips microarrays. Function and pathway enrichment analyses of statistically significant, differential expressed genes (*p* < 0.05) were performed using the Database for Annotation, Visualization and Integrated Discovery (DAVID) online tools.

**Results:**

*Helicobacter* infection affected the transcriptional profile of more genes in *Myd88* ^*−/−*^ mice compared to WT mice. Infection of *Myd88* ^*−/−*^ mice resulted in the differential expression of 1,989 genes at 25 weeks (1031 up and 958 downregulated). At 47 weeks post-*H.felis* infection, 2,162 (1140 up and 1022 downregulated) were differentially expressed. The most significant differentially upregulated gene during *Helicobacter* infection in *Myd88* ^*−/−*^ mice was chitinase-like 4 (chil4), which is involved in tissue remodeling and wound healing. Other highly upregulated genes in *H. felis*-infected *Myd88* ^−/−^ mice included, Indoleamine 2,3-Dioxygenase 1 (Ido1), Guanylate binding protein 2 (Gbp2), ubiquitin D (Ubd), β_**2**_-Microglobulin (B2m), CD74 antigen (Cd74), which have been reported to promote cancer progression by enhancing angiogenesis, proliferation, migration, metastasis, invasion, and tumorigenecity. For downregulated genes, the highly expressed genes included, ATPase H+/K+ transporting, alpha subunit (Atp4a), Atp4b, Mucin 5 AC (Muc5ac), Apolipoprotein A-1 (Apoa1), and gastric intrinsic factor (Gif), whose optimal function is important in maintaining gastric hemostasis and lower expression has been associated with increased risk of gastric carcinogenesis.

**Conclusions:**

These results provide a global transcriptional gene profile during the development and progression of *Helicobacter*-induced gastric cancer. The data show that our mouse model system is useful for identifying genes involved in gastric cancer progression.

**Electronic supplementary material:**

The online version of this article (doi:10.1186/s12885-017-3114-y) contains supplementary material, which is available to authorized users.

## Background

Gastric cancer is one of the most common causes of cancer-related death worldwide with an estimated 738,000 deaths each year [1]. Recently, *H. pylori* was recognized as the foremost cause of gastric cancer [2–7]. With an estimated half of the world’s population being infected, *Helicobacter* infection contributes significantly to the worldwide gastric cancer burden [7, 8]. Recognition of the factors leading up to the development and progression towards gastric cancer are critical in determination of cancer pathology. *H. pylori*-induced gastric carcinogenesis involves a multistep progression from normal gastric mucosa to superficial gastritis, chronic gastritis, atrophic gastritis, metaplasia, dysplasia, and finally gastric carcinoma [8, 9]. Molecular events associated with disease progression to gastric malignancy have not been elucidated. Considerable amount of confirmatory evidence shows that host immune response to *H. pylori* is crucial in determining gastric cancer predisposition [10–12]. We have previously shown that a key signal transduction adaptor protein, myeloid differentiation primary response gene 88 (MyD88), regulates *Helicobacter*-induced gastric cancer progression in a mouse model of gastric cancer [13]. We demonstrated that *H. felis*-infected MyD88 deficient (*Myd88* ^−/−^) mice exhibited severe gastric pathology and an accelerated progression to gastric dysplasia compared to wild type (WT) mice [13] However, the MyD88-dependent gene responsible for this pathology were not described.

MyD88 is a key adaptor molecule that is crucial in mediating innate immune signals from members of the toll-like receptor (TLR) and interleukin-1 (IL-1)/IL-18 families leading to downstream activation of nuclear factor (NF)-κB [14–16]. Consistent with involvement in these inflammatory pathways, MyD88 signaling has been associated with cancer progression, which stems from the understanding that inflammation is linked to cancer promotion [17, 18]. Studies on the role of MyD88 cancer progression have been the subject of recent intense investigations. However, the data are contradictory, which indicate that the role of MyD88 in the development and progression of inflammation-associated cancers is complex [19]. Several studies using genetic or chemical carcinogenesis models involving *Myd88* deficient mice have shown MyD88 to either promote [20–27] or suppress [13, 28–34] tumor development. The complex role of MyD88 in carcinogenesis is best typified by studies in colon cancer models [22, 24, 29, 35] showing contradictory roles in the same tissue. The mechanistic basis for these opposing observation is still not fully understood and could be due to many factors including, the type of inflammation, the extent of tissue damage, and immune response elicited [35]. Further, the MyD88 dependent genes in this accelerated progression to dysplasia remain unknown. Therefore, this study was performed to identify potential genes involved in the accelerated progression of gastric cancer.

## Results

### Gene expression and analysis

**Fig. 1:**
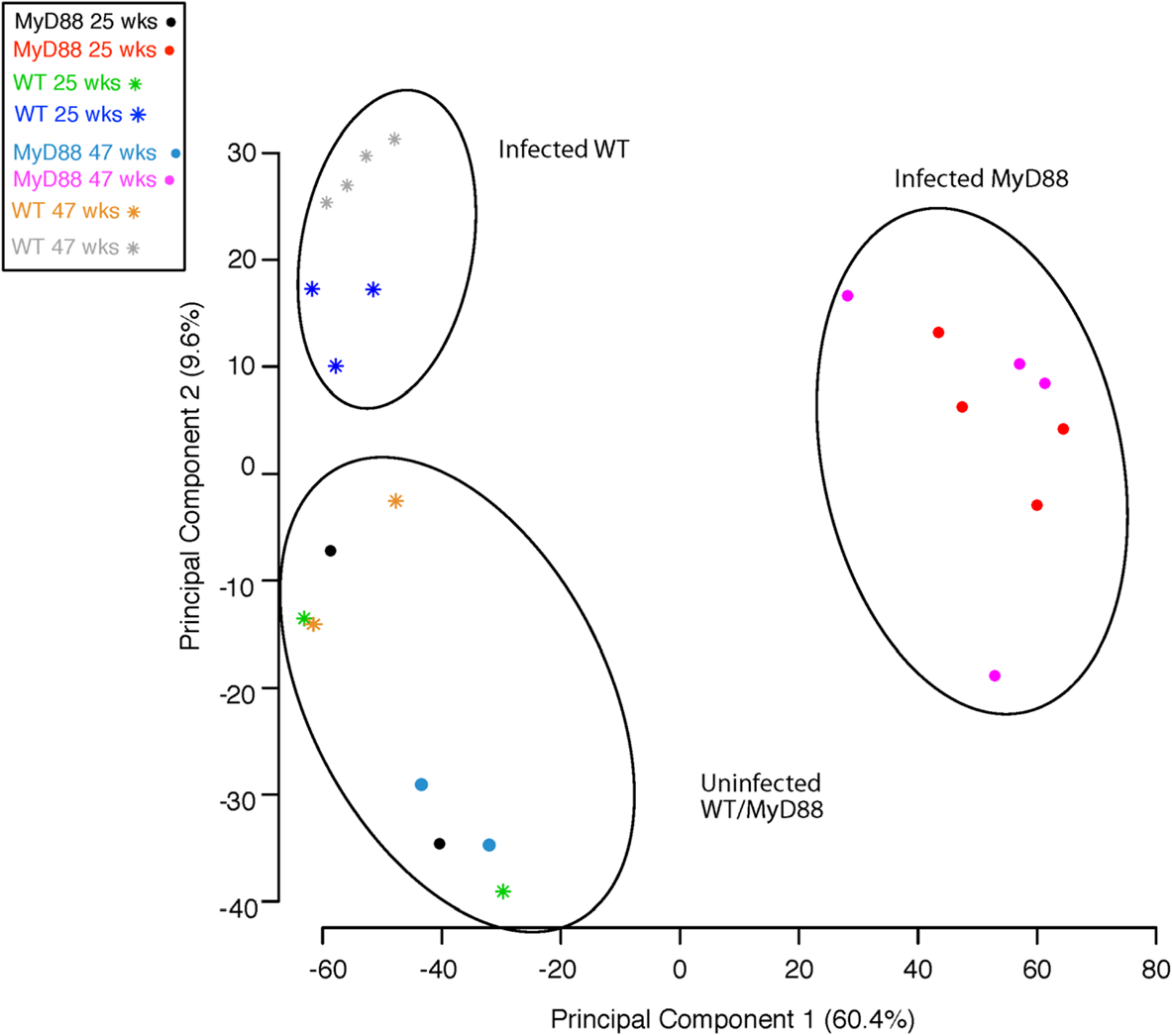
Multi Dimensional Scale Plot of all *H. felis* infected and control samples from WT and *Myd88* ^−/−^ mice. Samples were separated using 8 RNA SEQ libraries based on sample relations of 23,015 genes with a standard deviation/ mean >0.1 Groups separated into infected *Myd88* ^*−/−*^, infected WT and uninfected samples

Prior to differential gene analysis, all data from 23,015 genes with a standard deviation of less than 0.1 were used for multiple dimensional scaling (MDS) analysis (Fig. 1) to verify that *Myd88* ^*−/−*^ and WT samples were differentiated according to gene expression in each sample with a relative *p*-value. Each sample is represented with distance between each one reflecting their approximate degree of correlation [36]. All genes included in the analysis had a minimum standard deviation of less than 0.1. The analysis showed that all uninfected mice were clustered together irrespective of genetic background or time point. For infected mice, WT and *Myd88* ^−/−^ mice clustered distinctively separate indicating differential expression of their genes.

**Fig. 2:**
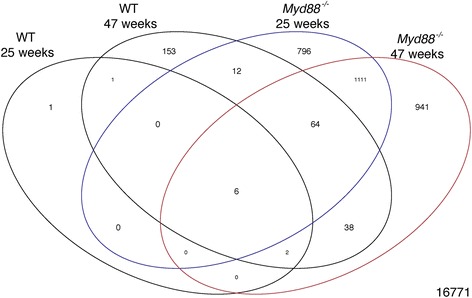
Venn diagram of differentially expressed genes. The number of changed genes following infection with *H. felis* in WT and *Myd88* ^*−/−*^ mice at 25 and 47 weeks (*p* < 0.05) is shown. The relationship between these DEGS is also shown

Statistical analysis of all 23,015 genes that went through the filtering process identified a total of 286 genes in WT and 4,151 in *Myd88* ^−/−^ mice in response to *H. felis* infection with more genes differentially expressed at 47 than 25 weeks (Table 1). Comparing the number of upregulated genes between *Myd88* ^*−/−*^ and WT at 47 weeks post infection, there were more upregulated genes (1140) in *Myd88* ^*−/−*^ mice compared to WT mice (189 genes). A similar trend was observed for upregulated genes at 25 weeks and for downregulated genes at both time points, with more genes differentially expressed in *Myd88* ^−/−^ than WT mice in response to *H. felis* infection. The number of differentiated genes at each time point in comparison to uninfected controls is illustrated in Fig. 2. Most genes overlapped between time points, however, there were a substantial number of genes that were unique to each set of time points that were differentially regulated (Fig. 2).

**Table 1:** Summary of microarray-based analysis of DEGs

Strain	UP	DOWN	TOTAL
WT 25 weeks	7	3	10
WT 47 weeks	189	87	276
*Myd88* ^*−/−*^ 25 weeks	1031	958	1989
*Myd88* ^*−/−*^ 47 weeks	1140	1022	2162

Number of differentially regulated genes at 25 weeks and 47 weeks in *Myd88* ^*−/−*^ and WT mice infected with *H. felis* compared to their matched uninfected controls. In total 23,015 genes were analyzed in both mouse backgrounds. Totals depict number of DEGs, both up and downregulated

**Fig. 3:**
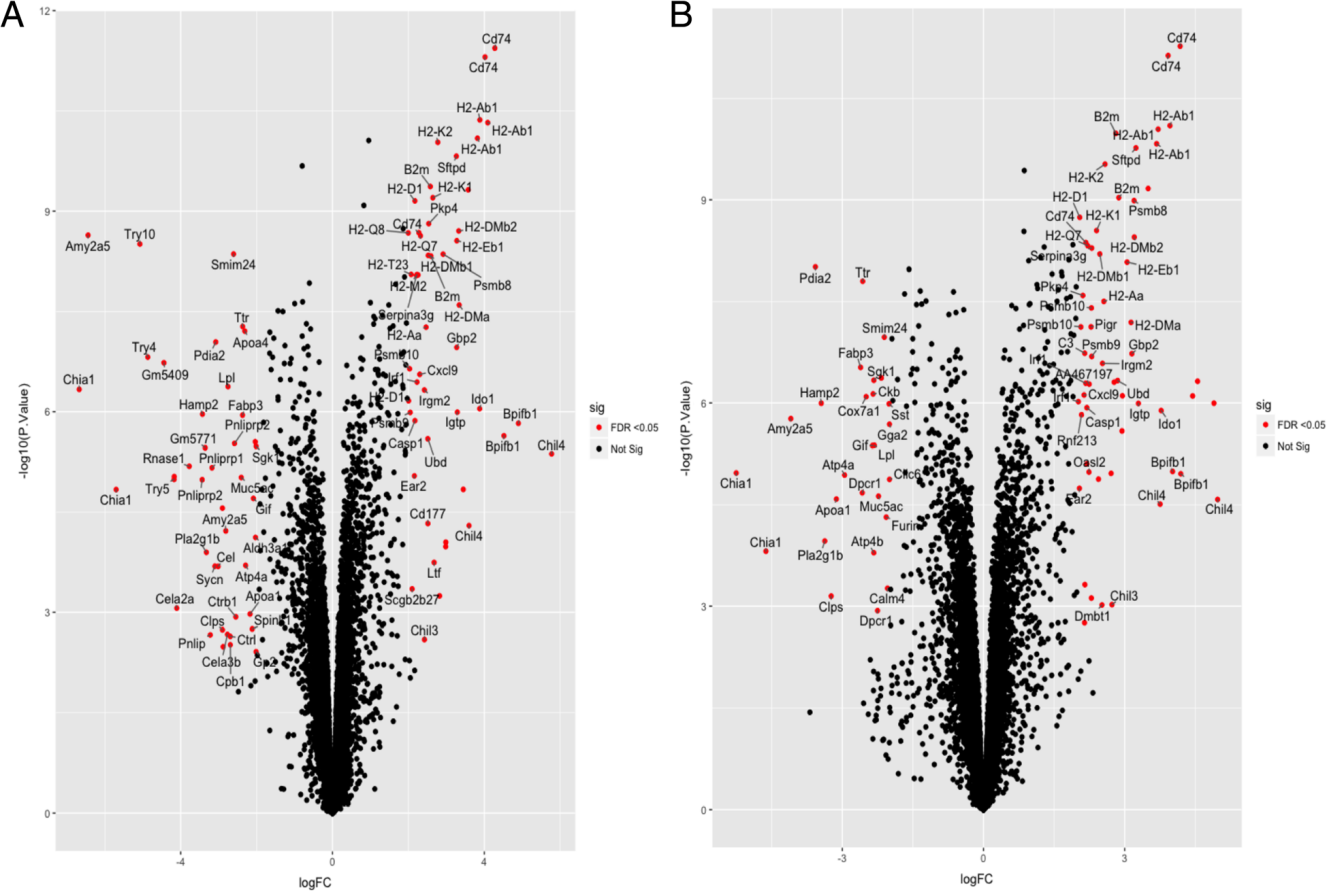
Scatterplot of Differentially Expressed Genes in *H. felis-* infected *Myd88* ^*−/−*^ samples at (**a**) 25 and (**b**) 47 weeks. Scatterplot represents a summary of t-tests for individual genes, depicting the Log_2_ fold changes and their corresponding –log_10_ *p*-values of all differentially expressed genes from microarray analysis. Genes were separated into different time points. Negative values of Log_2_ fold changes indicate downregulated genes. Positive Log_2_ fold changes indicate upregulated genes. Genes with a fold change < 2.0 and a *p* value <0.05 are depicted as red dots and genes not found to be significantly altered are depicted as black dots. All infected animals were normalized to uninfected control mice at the same time point

Scatterplot depiction of differentially expressed genes shows significantly (*P* < 0.05) up- and downregulated genes in *Myd88* ^*−/−*^ mice in response to *H. felis* infection (Fig. 3). A majority of genes were altered after 25 weeks of *H. felis* infection. Some of the new additional genes at 47 weeks post-*H. felis* infection included the ring finger protein 213 (Rnf213) and Furin, which have been reported to be involved in angiogenesis [37, 38] and cancer progression [39], respectively indicating their role in advanced stages of cancer progression.

### Analysis of differentially expressed genes

Tables 2 and 3 show a list of the top 50 up- and downregulated genes in *Myd88* ^−/−^ mice at 25 and 47 weeks post-*H. felis* infection compared to uninfected controls. The most highly upregulated gene during *H. felis* infection in *Myd88* ^*−/−*^ mice included Chitinase-like (chil4), which is involved in tissue remodeling and wound healing [40–42]. Many of the upregulated genes in both 25 weeks and 47 weeks post-*H. felis* infection involved genes in the H2 Complex (murine major MHC), particularly the class I heavy chains, H2-K and H2-D. The light chain for this MHC complex consists of the β_**2**_-Microglobulin (B2m) [43]. MHC class II antigen presentation including the CD74 antigen (Cd74) was another gene that was upregulated in response to *H. felis* infection. High expression of Cd74 has been linked to chronic inflammation and carcinogenesis in the gastrointestinal tract [44]. Another highly expressed gene was Indoleamine 2,3-Dioxygenase 1 (Ido1), which is suggested to play a role in immune tolerance and high expression in colorectal cancer and is correlated with a poor clinical outcome (reviewed in [45]. The entire list of altered genes in response to infection with *H. felis* including those in WT mice have been submitted and can be uploaded as an excel file in Additional files 1: Table S1 (inf vs uninfectd charts.xlsx).

**Table 2:** Top 50 most differentially expressed annotated genes in *H. felis*-infected *Myd88* ^*−/−*^ mice at 25 weeks compared to uninfected controls

Symbol	Gene Name	LogFC	Adj. P.Val
Chil4	chitinase-like 4	5.77	4.05E-04
Bpifb1	BPI fold containing family B, member 1	4.90	1.97E-04
Cd74	CD74 antigen (invariant polypeptide of major histocompatibility complex, class II antigen-associated)	4.28	4.93E-08
H2-Ab1	histocompatibility 2, class II antigen A, beta 1	4.09	2.35E-07
Ido1	indoleamine 2,3-dioxygenase 1	3.88	1.39E-04
H2-DMa	histocompatibility 2, class II, locus DMa	3.34	1.29E-05
H2-DMb2	histocompatibility 2, class II, locus Mb2	3.33	2.22E-06
Igtp	interferon gamma induced GTPase	3.29	1.54E-04
H2-Eb1	histocompatibility 2, class II antigen E beta	3.28	2.51E-06
Gbp2	guanylate binding protein 2	3.27	3.49E-05
Sftpd	surfactant associated protein D	3.26	3.73E-07
Psmb8	proteasome (prosome, macropain) subunit, beta type 8 (large multifunctional peptidase 7)	2.92	3.46E-06
H2-K2	histocompatibility 2, K region locus 2	2.78	2.64E-07
H2-K1	histocompatibility 2, K1, K region	2.64	1.04E-06
B2m	beta-2 microglobulin	2.59	3.46E-06
Pkp4	plakophilin 4	2.54	2.04E-06
H2-DMb1	histocompatibility 2, class II, locus Mb1	2.52	3.46E-06
Ubd	ubiquitin D	2.51	2.90E-04
H2-Aa	histocompatibility 2, class II antigen A, alpha	2.47	2.15E-05
Irgm2	immunity-related GTPase family M member 2	2.42	9.04E-05
H2-Q7	histocompatibility 2, Q region locus 7	2.32	2.22E-06
Cxcl9	chemokine (C-X-C motif) ligand 9	2.30	6.23E-05
Irf1	interferon regulatory factor 1	2.23	7.54E-05
Serpina3g	serine (or cysteine) peptidase inhibitor, clade A, member 3G	2.22	5.85E-06
H2-M2	histocompatibility 2, M region locus 2	2.21	5.85E-06
Casp1	caspase 1	2.17	1.86E-04
H2-D1	histocompatibility 2, D region locus 1	2.17	1.07E-06
Ear2	eosinophil-associated, ribonuclease A family, member 2	2.16	6.91E-04
H2-T23	histocompatibility 2, T region locus 23	2.07	5.85E-06
Psmb9	proteasome (prosome, macropain) subunit, beta type 9 (large multifunctional peptidase 2)	2.05	1.54E-04
Psmb10	proteasome (prosome, macropain) subunit, beta type 10	2.03	5.50E-05
Sgk1	serum/glucocorticoid regulated kinase 1	−2.01	3.48E-04
Apoa4	apolipoprotein A-IV	−2.31	2.33E-05
Ttr	transthyretin	−2.37	2.15E-05
Fabp3	fatty acid binding protein 3, muscle and heart	−2.37	1.61E-04
Muc5ac	mucin 5, subtypes A and C, tracheobronchial/gastric	−2.41	7.14E-04
Smim24	small integral membrane protein 24	−2.61	3.46E-06
Lpl	lipoprotein lipase	−2.75	8.29E-05
Pdia2	protein disulfide isomerase associated 2	−3.08	3.10E-05
Pnliprp1	pancreatic lipase related protein 1	−3.18	5.76E-04
Gm5771	predicted gene 5771	−3.36	3.53E-04
Hamp2	hepcidin antimicrobial peptide 2	−3.43	1.59E-04
Pnliprp2	pancreatic lipase-related protein 2	−3.44	7.47E-04
Rnase1	ribonuclease, RNase A family, 1 (pancreatic)	−3.78	5.55E-04
Try5	trypsin 5	−4.17	7.45E-04
Gm5409	predicted pseudogene 5409	−4.44	4.89E-05
Try4	trypsin 4	−4.87	4.39E-05
Try10	trypsin 10	−5.08	2.69E-06
Amy2a5	amylase 2a5	−6.44	2.22E-06
Chia1	chitinase, acidic 1	−6.68	8.93E-05

Genes with no known annotated name were excluded from the analysis. Genes were considered to be statistically significant when a threshold adjusted *p* value < 0.05 and Log FC > 2 were reached

**Table 3:** Top 50 most differentially expressed annotated genes in *H. felis*-infected *Myd88* ^*−/−*^ mice at 47 weeks compared to uninfected controls

Gene Symbol	Gene Name	LogFC	Adj. P.Val
Chil4	chitinase-like 4	4.97	1.24E-03
Cd74	CD74 antigen (invariant polypeptide of major histocompatibility complex, class II antigen-associated)	4.18	7.41E-08
Bpifb1	BPI fold containing family B, member 1	4.02	6.43E-04
H2-Ab1	histocompatibility 2, class II antigen A, beta 1	3.96	4.14E-07
Ido1	indoleamine 2,3-dioxygenase 1	3.78	1.44E-04
Igtp	interferon gamma induced GTPase	3.29	1.25E-04
Sftpd	surfactant associated protein D	3.24	4.86E-07
H2-DMb2	histocompatibility 2, class II, locus Mb2	3.20	4.43E-06
Psmb8	proteasome (prosome, macropain) subunit, beta type 8 (large multifunctional peptidase 7)	3.20	1.71E-06
Gbp2	guanylate binding protein 2	3.15	4.53E-05
H2-DMa	histocompatibility 2, class II, locus DMa	3.13	2.17E-05
H2-Eb1	histocompatibility 2, class II antigen E beta	3.05	6.39E-06
Ubd	ubiquitin D	2.85	8.22E-05
B2m	beta-2 microglobulin	2.81	4.14E-07
H2-K2	histocompatibility 2, K region locus 2	2.58	7.37E-07
H2-Aa	histocompatibility 2, class II antigen A, alpha	2.56	1.39E-05
Irgm2	immunity-related GTPase family M member 2	2.53	5.62E-05
H2-DMb1	histocompatibility 2, class II, locus Mb1	2.47	5.72E-06
H2-K1	histocompatibility 2, K1, K region	2.40	3.89E-06
Serpina3g	serine (or cysteine) peptidase inhibitor, clade A, member 3G	2.30	4.90E-06
Psmb9	proteasome (prosome, macropain) subunit, beta type 9 (large multifunctional peptidase 2)	2.30	4.92E-05
Psmb10	proteasome (prosome, macropain) subunit, beta type 10	2.29	1.49E-05
Pigr	polymeric immunoglobulin receptor	2.29	2.37E-05
Oasl2	2′-5′ oligoadenylate synthetase-like 2	2.24	6.52E-04
H2-Q7	histocompatibility 2, Q region locus 7	2.22	4.90E-06
Casp1	caspase 1	2.20	1.33E-04
Irf1	interferon regulatory factor 1	2.17	8.33E-05
C3	complement component 3	2.15	4.52E-05
Cxcl9	chemokine (C-X-C motif) ligand 9	2.14	1.04E-04
Pkp4	plakophilin 4	2.11	1.28E-05
Rnf213	ring finger protein 213	2.08	1.59E-04
H2-D1	histocompatibility 2, D region locus 1	2.04	2.78E-06
Ear2	eosinophil-associated, ribonuclease A family, member 2	2.04	9.57E-04
Sst	somatostatin	−2.01	1.25E-04
Smim24	small integral membrane protein 24	−2.11	3.12E-05
Muc5ac	mucin 5, subtypes A and C, tracheobronchial/gastric	−2.23	1.14E-03
Lpl	lipoprotein lipase	−2.32	3.50E-04
Sgk1	serum/glucocorticoid regulated kinase 1	−2.33	8.22E-05
Ckb	creatine kinase, brain	−2.35	1.02E-04
Gif	gastric intrinsic factor	−2.36	3.50E-04
Cox7a1	cytochrome c oxidase subunit VIIa 1	−2.49	1.06E-04
Ttr	transthyretin	−2.57	9.85E-06
Dpcr1	diffuse panbronchiolitis critical region 1 (human)	−2.57	1.05E-03
Fabp3	fatty acid binding protein 3, muscle and heart	−2.61	6.23E-05
Atp4a	ATPase, H+/K+ exchanging, gastric, alpha polypeptide	−2.95	6.99E-04
Apoa1	apolipoprotein A-I	−3.13	1.23E-03
Hamp2	hepcidin antimicrobial peptide 2	−3.45	1.25E-04
Pdia2	protein disulfide isomerase associated 2	−3.57	7.21E-06
Amy2a5	amylase 2a5	−4.09	1.74E-04
Chia1	chitinase, acidic 1	−5.25	6.66E-04

Genes with no known annotated name were excluded from the analysis. Genes were considered to be statistically significant when a threshold adjusted *p* value < 0.05 and Log FC > 2 were reached

**Fig. 4:**
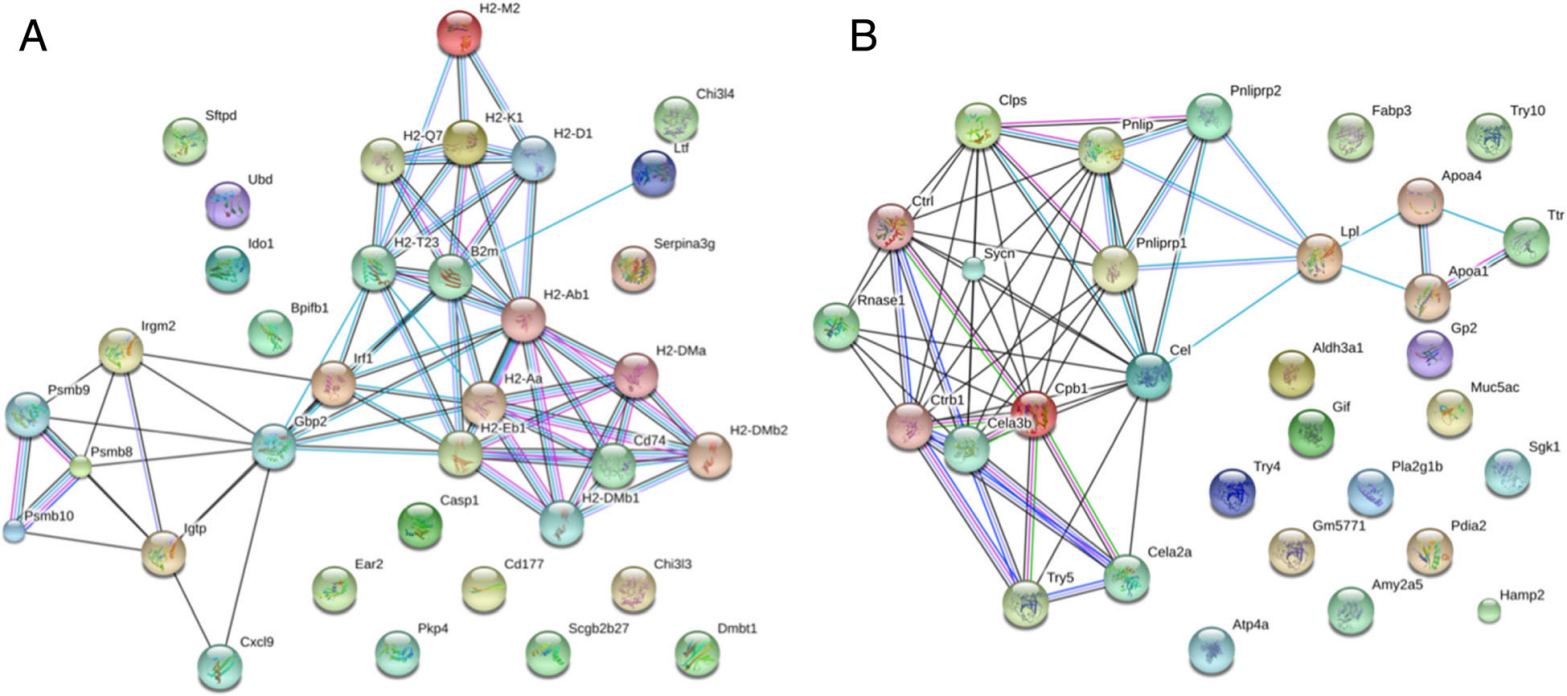
Network characterization of selected genes at 25 weeks. STRING gene networks of interactions of DEGs with a STRING interaction confidence of 0.7 or greater (high confidence) for *H. felis-*infected *Myd88* ^*−/−*^ mice at 25 weeks for both upregulated (**a**) and downregulated (**b**) genes. Known interactions are illustrated with light blue string attachments (from curated databases) and light pink/purple strings (experimentally determined). Co-expression are illustrated with black string attachments

**Fig. 5:**
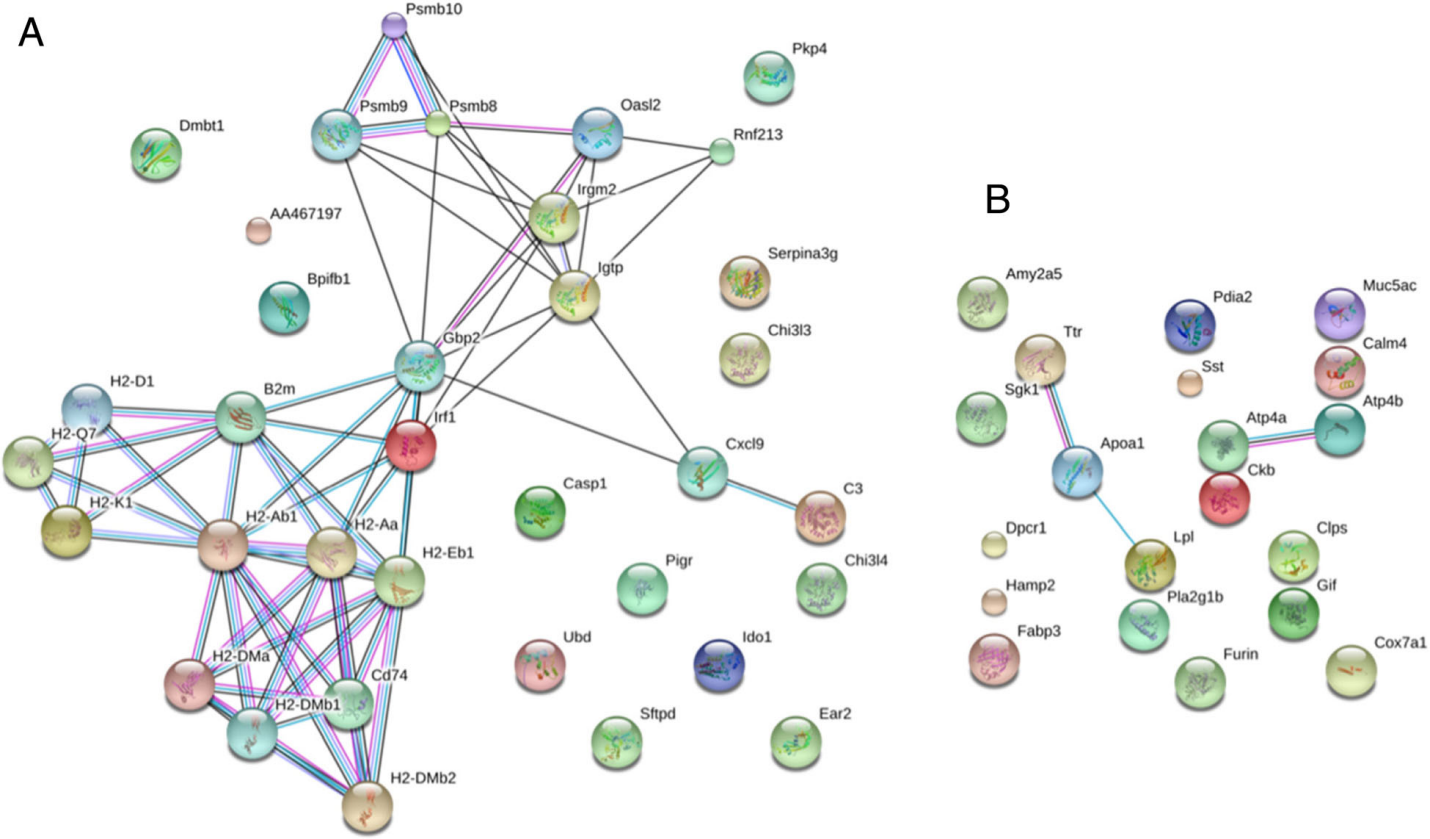
Network characterization of selected genes at 47 weeks. STRING gene networks of interactions of DEGs with a STRING interaction confidence of 0.7 or greater (high confidence) for *MyD88* ^*−/−*^ *- H. felis* infected at 47 weeks both upregulated (**a**) and downregulated (**b**) genes. Known interactions are illustrated with light blue string attachments (from curated databases) and light pink/purple strings (experimentally determined). Co-expression are illustrated with black string attachments

STRING summary networks depicting protein- protein interactions among the top differentially expressed genes (DEGs) for both up- and downregulated genes in *Myd88* ^−/−^ mice are shown in Figs. 4 and 5 for 25 and 47 weeks, respectively. Thicker lines connecting the genes indicate a stronger association between the genes. A confidence score of at least 0.70 (high) was used. One of the key central nodes in the top DEGs in *Myd88* ^−/−^ mice at both 25 and 47 weeks post-infection was guanylate-binding protein 2 (Gbp2), which is considered a potential marker for esophageal squamous cell carcinoma [46].

### Functional enrichment analysis of differentially expressed genes (DEGs)

**Fig. 6:**
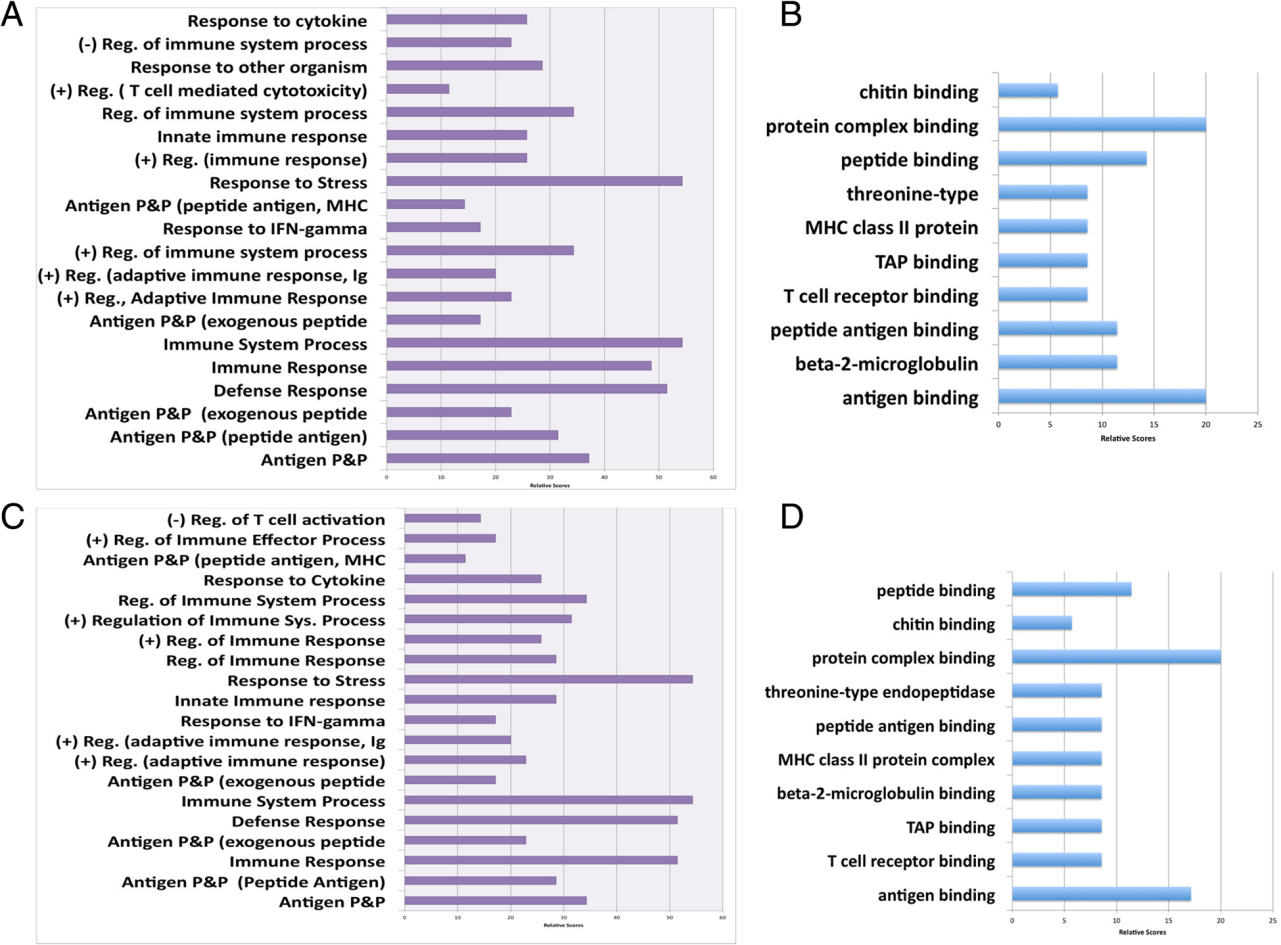
Profiles of GO enrichment analysis. Enriched Go terms are shown for *H. felis*-infected *Myd88* ^*−/−*^ mice at both 25 (**a**, **b**) and 47 weeks (**c**, **d**). Biological processes are depicted in figures A and C while molecular functions are depicted in B and D. For the biological processes, the top 20 processes are shown. All scores depicted are relative scores for number of genes in each function/ process relative to the number of total genes entered into the STRING database

**Fig. 7:**
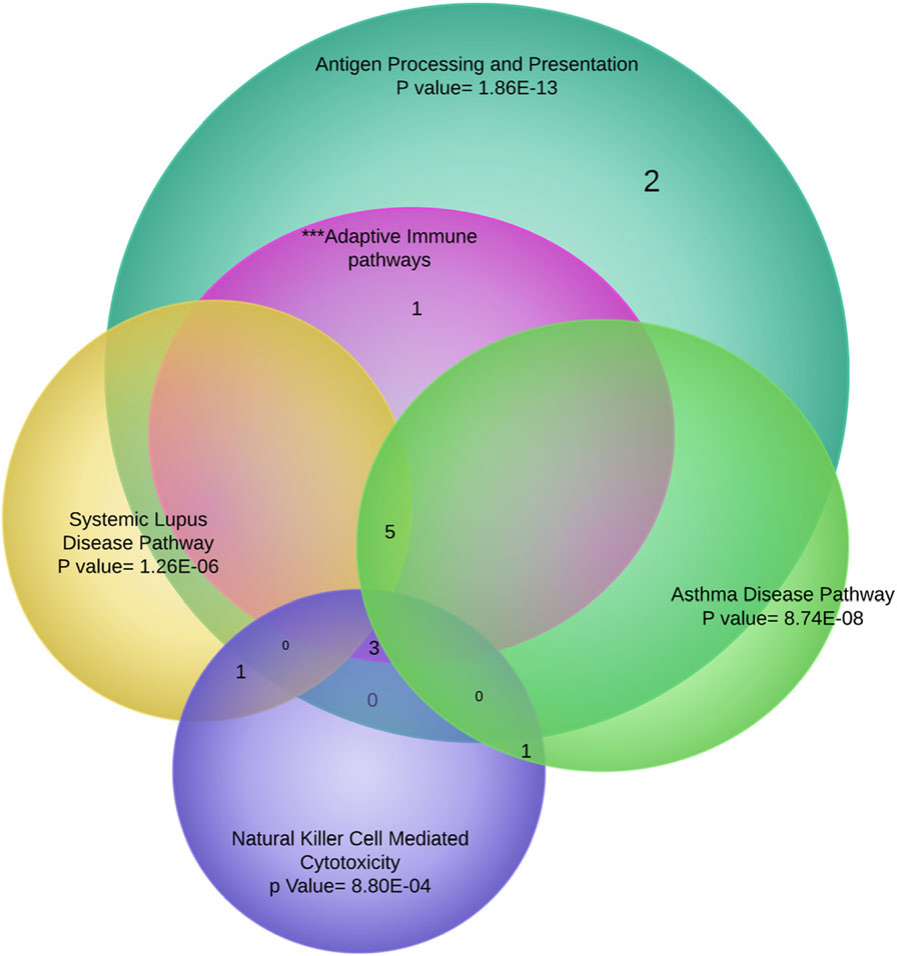
Venn Diagram showing distribution of different significantly enriched KEGG pathways. Pathways with *p*-value > E-04 are included. Antigen processing and presentation is the most enriched pathway overlapping the majority of genes with all other pathways. Notably, all processes are associated with inflammatory responses of the immune system

To gain insights into the biological meaning and function of the differentially expressed genes, enrichment analysis was performed using the database for annotation, visualization and integrated discovery (DAVID) online analytical tools [47–49]. Annotation according to tissue expression, molecular function, cellular component and biological processing was done using Gene Ontology (GO) [49]. Enrichment analysis was performed to identify pathways, processes and gene categories that are over-represented in the list of DEGs compared to the mouse genome. GO clustering analysis for biological processes showed that responses related to immune system processes were the most upregulated enriched process in *Myd88* ^−/−^ mice at both 25 and 47 weeks in response to *H. felis* infection (Fig. 6a and c). For molecular functions, antigen and protein complex binding were the most enriched processes (Fig. 6b and d). Downregulated enriched processes in *Myd88* ^−/−^ mice in response to *H. felis* infection are presented as supplementary data (Additional files 2: Figure S1). A summary of the Kyoto Encyclopedia of Genes and Genomes (KEGG) pathway annotation shown in Fig. 7 revealed that the most enriched pathway was antigen processing and presentation. This pathway shared the majority of its genes with the other major pathways and completely engulfed the other pathways by sharing more than 90% of the genes annotated. A breakdown of up- and downregulated KEGG pathway at 25 and 47 weeks in *Myd88* ^−/−^ mice in response to *H. felis* infection is presented as supplementary data (Additional files 3: Figure S2).

## Discussion

Gastric cancer develops and progresses through a stepwise sequence of events from inflammation to atrophy, metaplasia, dysplasia, and finally to gastric cancer [50]. We previously demonstrated using a mouse model of gastric that mice deficient in MyD88 signaling exhibited dramatic pathology and an accelerated progression to gastric neoplasia in response to *H. felis* infection [13]. In the present study, we used microarray gene expression analysis to identify the genes involved in this progression to gastric neoplasia. Although previous studies have investigated differential gene expression in mice stomachs in response to *Helicobacter* infection, most have focused on *H. pylori* [51–53], which does not result in neoplastic changes in mice [54, 55]. The few studies that have examined gene expression profiles in mouse model of gastric cancer have used the insulin-gastrin (INS-GAS) transgenic gastric cancer mouse model [56, 57]. These mice have been shown to spontaneously develop gastric cancer even in the absence of *Helicobacter* infection [58]. We have previously reported that *Myd88* ^−/−^ mice do not exhibit abnormal pathology in the absence of *Helicobacter* infection [13]. The global transcriptional profiling of mouse gastric tissue identified a large number of significant differentially expressed genes in *H. felis*-infected *Myd88* ^−/−^ mice compared to *H. felis*-infected WT mice. The most over expressed gene in *Myd88* ^*−/−*^ mice during *H. felis* infection at 25 weeks was Chil4. Chitinase like proteins (CLPs) have been studied in relation to other cancers yet little has been investigated in relation to gastric cancer except for our present study and a couple other studies [57, 59]. Upregulation of CLPs has been shown in a number of human cancers including brain, bone, breast, ovaries, lung, prostate, colon, thyroid, and liver [41, 60]. For gastric cancer studies, chitinase protein 3 like 1 (Chil1) was upregulated in INS-GAS mice infected with *H. felis*, [57]. In our present study, Chil1 was not upregulated in response to *H. felis* infection. However, in addition to upregulation of Chil4, another CLP, Chil3 was also significantly over expressed in *H. felis*-infected mice at both 25 weeks (*p* = 0.03) and 47 weeks (*p* = 0.01) (gene not listed in Tables 2 and 3, only the top 50 are listed). An abundant over expression of Chil1, Chil4 as well as Chil3 has been reported in early preneoplastic stage in the epidermis [61]. Overall, CLPs have been implicated to play a role in chronic inflammation, tissue remodeling, and wound healing [40]. Up-regulation of genes involved in tissue remodeling is noteworthy because chronic inflammation and subsequent damage to the gastric epithelium has been suggested to play an important role in cancer development and progression [62]. During chronic inflammation, the resulting prolonged tissue damage creates a loss of control over normal tissue repair mechanisms resulting in persistent hyper-tissue repair, which is accompanied with sustained proliferation [63] and ultimately advancing to precancerous lesions. Lost tissue is then replaced with stem and progenitor cells that are under a continuous stimulus of proliferation, leading to the accumulation of replacement cells with dysregulated and altered signaling pathways [63]. Further, studies investigating associations between chronic inflammation, tissue repair and carcinogenesis highlight the potential of these cellular changes in inducing both pro-oncogenic and tumor suppressor pathways [62, 64–67]. Our study, in addition to the work done by Li et al. [59] and Takaishi and Wang [57] show a need for further investigation into the role of CLPs in gastric carcinogenesis.

Other upregulated genes included Cd74, B2m, and interferon (IFN) induced genes such as GTPases (interferon gamma induced GTPase, lgtp, immune mediated GTPase family M member 2, lrgm2), Guanylate binding protein 2 (Gbp2), and transcription factor interferon regulatory factor 1 (Irf1). Cd74 or invariant chain (Ii) protein is a chaperone molecule responsible for regulating antigen presentation of MHC II molecules. It has been linked to chronic inflammation and carcinogenesis in the gastrointestinal tract [44]. Further, Cd74 was also shown to play a role as a receptor for migration inhibitory factor (MIF), a molecule reported to have pro-carcinogenic effects on gastric epithelial cells [68]. IFNs are known to activate signal transducer and activator of transcription 3 (STAT3) [69, 70] signaling leading to epithelial proliferation and inhibition of apoptosis [71, 72]. Currently not much is known about IFNs in gastric cancer. Ubiquitin D (Ubd), which is associated with progression of colon cancer [73], was also significantly expressed genes in *Myd88* ^−/−^ in response to *H. felis* infection.

For downregulated genes, the significantly expressed ones included, ATPase H+/K+ transporting, alpha subunit (Atp4a), Atp4b, Mucin 5 AC (Muc5ac), apolipoprotein A-1 (Apoa1), and gastric intrinsic factor (Gif). The genes, Atp4a and Atp4b encode gastric H+/K + − ATPase alpha and beta subunits, respectively. Gastric H+/K + − ATPase alpha and beta subunits are expressed in parietal cells [74] and their loss has been associated with gastric dysplasia [58]. We observed downregulation of Atp4a and Atp4b in response to infection with *H. felis*, which may represent a loss of parietal cells that has been shown to precede gastric dysplasia. These results are in line with those observed in another fast progressing gastric cancer model involving the use of INS-GAS mice [58]. Muc5ac, which encodes gastric M1 mucin [75] has been reported to play a role in gastric carcinogenesis [76] was also downregulated in response to infection with *H. felis* in *Myd88* ^−/−^ mice. Progression of gastric lesions has been reported to be associated with the gradual decrease in expression of Muc5ac [57, 77–79] followed by the transformation of the gastric epithelium [80] resulting in gastric dysplasia. Another highly downregulated gene we found in *Myd88* ^−/−^ mice infected with *H. felis* was Apoa1, which was also reported to be downregulated in a fast progressing gastric cancer mouse model [57]. Proteomics approach in human gastric cancer also showed downregulation of *Apoa1* [81], but its role in gastric carcinogenesis is unknown. In the lung, downregulation of *Apoa1* was associated with an increased risk of lung cancer [82]. Data from *Apoa1*-deficient mice suggest antitumorigenic properties of *Apoa1* via modulation of the immune system [83]. Gastric intrinsic factor (Gif), another downregulated gene is secreted by parietal cells and is required for Vitamin B12 absorption [84]. Concomitantly, the downregulation of Gif results in vitamin B12 deficiency (pernicious anemia). Gastric intrinsic factor was downregulated in *H. felis*-infected *Myd88* ^−/−^ mice at both 25 and 47 weeks post-infection. A previous study using INS-GAS mice infected with *H. felis* also reported downregulation of Gif [57]. In human gastric cancer, Gif was one of the genes found by SAGE analysis to be downregulated [85]. Further, studies have shown an increased risk of gastric cancer in pernicious anemic patients [86].

We found a number of new genes in our fast progressing gastric mouse model, i.e., *Myd88* ^−/−^ mice infected with *H. felis*. The genes included up- and downregulated genes, which had not been previously linked to *Helicobacter*-related gastric carcinogenesis including BPI fold containing family B member 1 (Bpifb1) and proteasome subunit beta 8 (Psmb8). These genes have been linked to cancer-related processes including, apoptosis and in some cases other cancers as well as prognosis indicators [87, 88]. Psmb8 was shown to be significantly up regulated in cancers such as bladder, breast, kidney, lung, uterine, and head and neck [89]. A recent study by Kwon, et al. [90], which was published during the writing of our manuscript reported that Psmb8 may be a potential marker for prognosis in gastric cancer. Bpifb1 may be involved in the innate immune response particularly in response to bacterial exposure. The protein encoded for by Bpifb1 binds bacterial lipopolysaccharide (LPS) as well as modulates the cellular response to LPS [91]. Bpifb1 has been found to be overexpressed in mucous cells of salivary gland tumors of papillary cystadenocarcinoma [87]. Future studies using a gastric culture organoid system will validate these genes and some of the important novel genes we identified for their role in rapid progression of *Helicobacter*-induced gastric cancer.

## Conclusions

In this study, we have identified genes that are involved in the rapid progression of *Helicobacter*-induced gastric cancer that are also potentially regulated by MyD88. The identification of these important genes could potentially serve as targets for disease prevention. In addition, we show that our model is a useful mouse model system to identify genes involved in gastric cancer progression.

## Methods

### Animals

Six- to ten- week-old wild type (WT) and MyD88 deficient (*Myd88* ^−/−^) mice in the C57BL/6 background were used in this study. WT mice were purchased from The Jackson Laboratory (Bar Harbor, ME). *Myd88* ^−/−^ mice were from our breeding colony originally provided by Dr. Akira (Osaka University, Japan). All mice were housed together before infection with *H. felis* and for the duration of the study. The Institutional Animal Care and Use Committee at the University of California, San Diego, approved all animal procedures and performed using accepted veterinary standards.

### Bacterial growth conditions

*Helicobacter felis*, strain CS1 (ATCC 49179) was purchased from American Type Culture Collection (Manassas, VA). *H. felis* was routinely maintained on solid medium, Columbia agar (Becton Dickinson, MD) supplemented with 5% laked blood under microaerophilic conditions (5% O_2_, 10% CO_2_, 85% N_2_) at 37 °C and passaged every 2–3 days as described previously [13, 92]. Prior to mouse infections, *H. felis* was cultured in liquid medium, brain heart infusion broth (BHI, Becton Dickinson) supplemented with 10% fetal calf serum and incubated at 37 °C under microaerophilic conditions for 48 h. Spiral bacteria were enumerated using a Petroff-Hausser chamber before infections.

### Mouse infections

A well-characterized cancer mouse model, which involves infecting C57BL/6 mice with *H. felis* (strain CS1), a close relative of the human gastric pathogen *H. pylori* was used in this study. Mice were inoculated with 10^9^organisms in 300 μL of BHI by oral gavage three times at 2-day intervals as previously described [13, 92]. Control mice received BHI only. At 25 and 47 weeks post-infection, mice were euthanized and the stomachs removed under aseptic conditions and processed for assessment of gene expression.

### RNA extraction and oligonucleotide microarray hybridization

Total RNA was extracted from gastric tissue obtained from *H. felis*-infected and uninfected WT and *Myd88* ^*−/−*^ mice. Stomach tissue sections of 12 mice i.e., two uninfected controls and four infected WT (6) and *MyD88* ^*−/−*^ (6) per time point (25 and 47 weeks) were analyzed. RNA was extracted from each section using the RNeasy miniprep kit (Qiagen) according to the manufacturer’s instructions followed by digestion with DNase 1 to remove genomic DNA. RNA concentration was determined using a NanoDrop spectrophotometer (NanoDrop Technologies, Inc., Waltham, MA). Double stranded cDNA and biotin-labeled cRNA were synthesized following the recommended Illumina protocol. Integrity of purified cRNAs was assessed on an Agilent 2100 Bioanalyzer prior to hybridization. 1.5 *μ*g of labeled cRNA was hybridized to MouseWG-6 v2 Expression BeadChips genome wide arrays, which analyzes 25,600 transcripts (Illumina, San Diego, CA) using recommended Illumina reagents and protocols.

### Identification of differentially expressed genes

Probe profiles (each row corresponding to a given probe and different columns for each sample) were exported from Genome Studio v1.8 (Illumina). The resultant tab-delimited file (Additional file 4, Probe_Profile_f.txt) was used as input for the Bioconductor *lumi* v2.18 R package (http://bioconductor.org/packages/release/bioc/html/lumi.html) [93]. Sample information is provided in Additional file 5 (sampleInfotxt.txt). Following quality assessment [density plot of intensity, cumulative density (CD), MA (transformed data onto log ratios and mean average) and pairwise MDS plots], the data were transformed using the *vst* (variance-stabilizing transformation) algorithm [94] then normalized using the Robust Spline Normalization (*rsn)* algorithm [94]. A second round of quality control of the normalized data was done to ensure data quality. After normalization genes with minimal variance across samples and genes that were not expressed in any sample (determined by detection calls) were removed prior to differential gene expression using linear models as implemented in *limma* [95]. We compared gene expression profiles of uninfected and infected mice at 25 and 47 weeks post infection. All mice used were on the C57BL/6 background. In addition, after normalization, significantly expressed genes were identified through volcano plot generation on the R - platform. Genes exhibiting statistically significant differential mean expression values (*p* < 0.05) were subjected to hierarchical clustering.

### Enrichment analysis and pathway generation

To analyze KEGG pathway enrichment, official gene symbols for differentially expressed genes were submitted to DAVID (Database for Annotation, Visualization and Integrated Discovery) (http://david.abcc.ncifcrf.gov). This tool allows for identification of over expression within a set of genes as compared to the entire genome of a specific animal [47]. In addition, it allows for the demonstration of Gene Ontology (GO) terms as well as pathway and functional processes (molecular functions, biological expression, and cellular expression) enrichment. Functionally enriched gene networks, KEGG Pathways and ontology terms were identified. The mouse, *Mus musculus* complete genome was used as background genes. All functionally annotated genes presented in gene networks or pathways had *p* values < 0.05.

### Gene network analysis

Gene networks were built using data on protein-protein interactions from EMBL STRING (Search Tool for the Retrieval of Interacting Genes/Proteins) database (http://string-db.org). This search tool is used to identify interactions correlated to expression data and/or literature citations among other criteria [96]. All gene connections created using STRING had a combined confidence score higher than 0.7 as previously described [97], Garcia-Alonso, 2014 #613}.

### Statistical analysis

Statistics were done on the R platform using the Limma package from Bioconductor. To control for multiple testing, the False Discovery Rate (FDR) method was used with a cutoff for statistical significance of *P* values of < 0.05 and a log fold expression of 2. Differentially expressed genes were determined at 25 and 47 weeks after removing background differences from both *Myd88* ^*−/−*^ and WT mice by comparing infected to uninfected mice in the same background.

## Additional files

Additional files 1:Table S1. Excel file: Data of upregulated and downregulated genes in *Myd88* ^−/−^ and WT mice at 25 and 47 weeks. (XLSX 4782 kb)
Additional files 2:Figure S1. Downregulated biological processes and molecular functions in *Myd88* ^−/−^ mice. Enriched Go terms are shown at both 25 (A, B) and 47 weeks (C, D). Biological processes are depicted in figures A and C while molecular functions are depicted in B and D. These functions were identified using STRING functional annotation tool. Relative scores were calculated using the number of genes found within each process relative to the total number of genes entered into the annotation tool. The top 20 Biological Processes are shown. (PPTX 482 kb)
Additional files 3:Figure S2. KEGG Pathways for both up and downregulated Genes at 25 and 47 weeks in *Myd88* ^−/−^ mice. KEGG pathway analysis of up- and downregulated genes in *Myd88* ^−/−^ mice at 25 (Additional files 3: Figure S2a and b) and 47 weeks (Additional files 3: Figure S2c and d). Significantly enriched pathways (*p* < 0.05) are presented in each pie graph. (PPTX 1888 kb)
Additional files 4: Text File: Probe Profile of Microarray Data used in analysis of gene expression. (TXT 30459 kb)
Additional files 5: Text file: Sample Info file listing name and ID of each mouse used in the analysis. (TXT 533 bytes)

## Abbreviations

(NF)-κB: Nuclear factor kappa B
Apoa1: Apolipoprotein A-1
Atp4a: ATPase H+/K+ alpha subunit
B2m: β_**2**_-microglobulin
Cd74: CD74 antigen
Chil4: Chitinase-like 4
DAVID: Database for Annotation, Visualization and Integrated Discovery
Gbp2: Guanylate binding protein 2
Gif: Gastric intrinsic factor
GO: Gene ontology
Ido1: Indoleamine2, 3- dioxygenase 1
IFN: Interferon
IL-1/IL-8: Interleukin-1/-8
INS-GAS: Insulin-gastrin transgenic gastric mice
KEGG: Kyoto Encyclopedia of Genes and Genomes
MDS: Multi dimensional scaling
MIF: Migration inhibitory factor
Muc5ac: Mucin 5 AC
Rnf213: Ring finger protein 213
STAT3: Signal transducer and activator of transcription 3
TLR: Toll like receptor
Ubd: Ubiquitin d
