# Supplementary figures and images for: Effect of myeloid differentiation primary response gene 88 on expression profiles of genes during the development and progression of Helicobacter-induced gastric cancer
Source: BMC Cancer. 2017 Feb 15;17:133. doi: 10.1186/s12885-017-3114-y (PMC5310019; doi:10.1186/s12885-017-3114-y)

## Slide 1
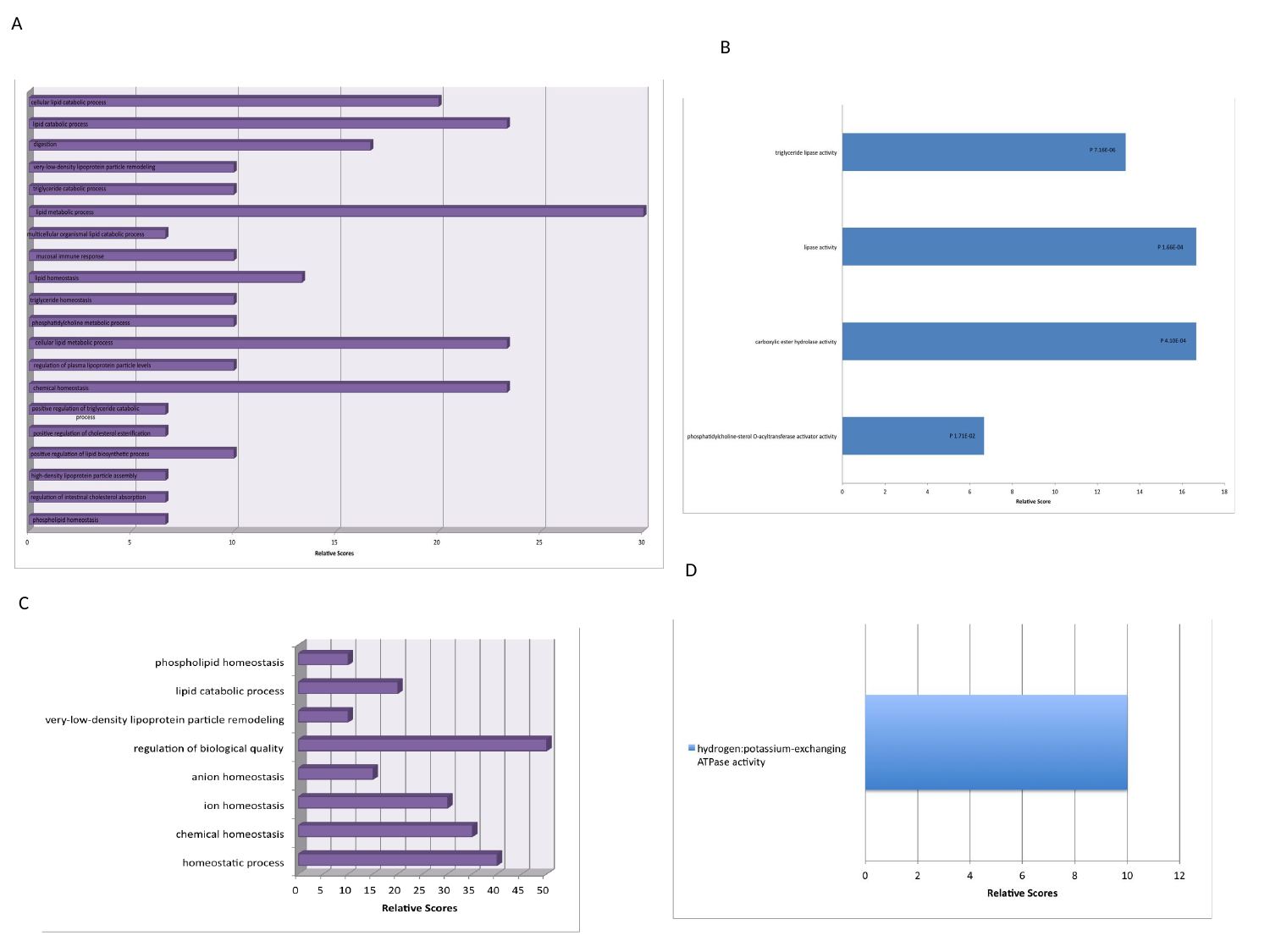

A
B
D
C

Supplement: Additional files 2:Figure S1. — Downregulated biological processes and molecular functions in Myd88 −/− mice. Enriched Go terms are shown at both 25 (A, B) and 47 weeks (C, D). Biological processes are depicted in figures A and C while molecular functions are depicted in B and D. These functions were identified using STRING functional annotation tool. Relative scores were calculated using the number of genes found within each process relative to the total number of genes entered into the annotation tool. The top 20 Biological Processes are shown. (PPTX 482 kb) [file 12885_2017_3114_MOESM2_ESM.pptx]

## Slide 1
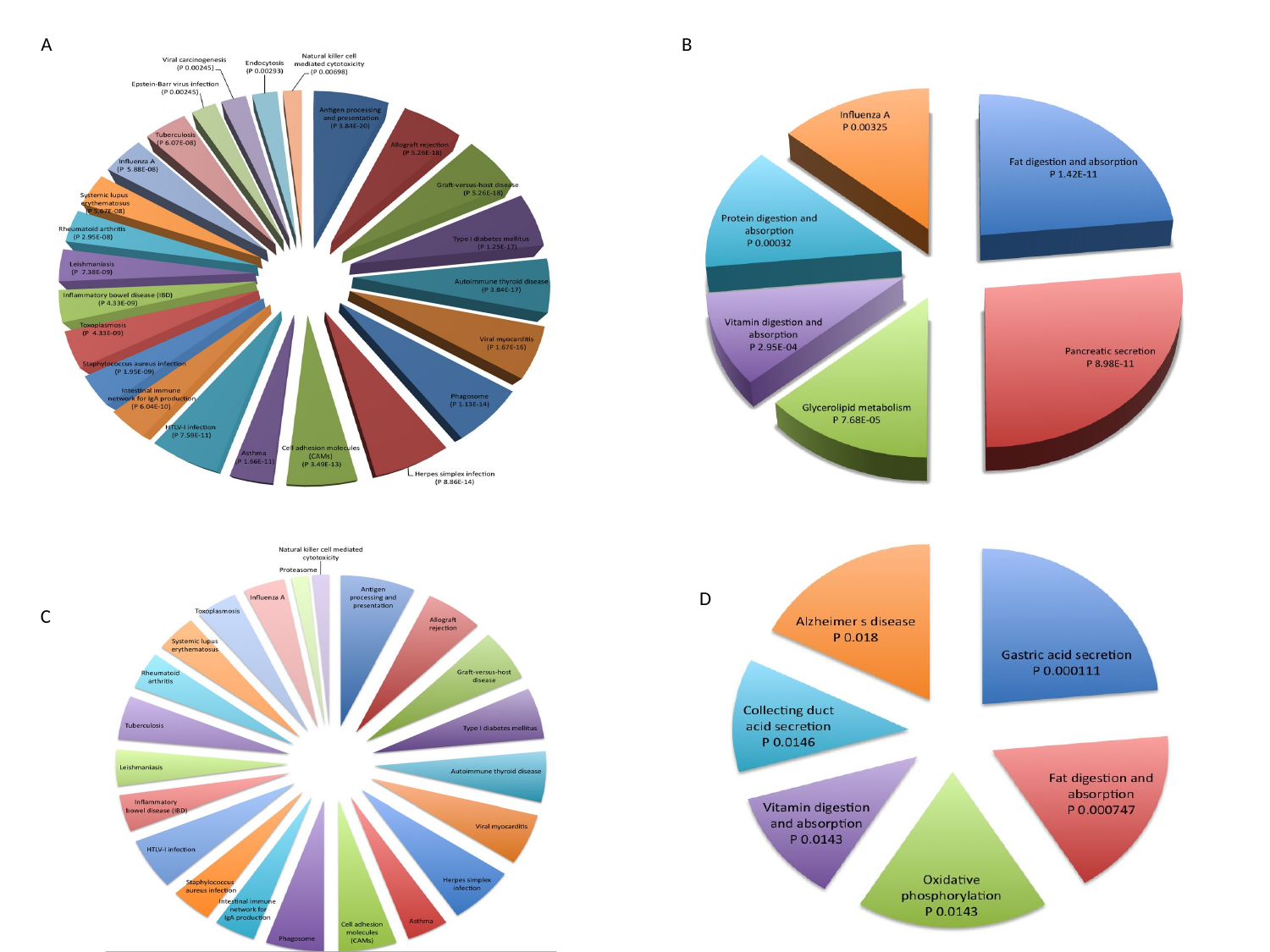

A
B
D
C

Supplement: Additional files 3:Figure S2. — KEGG Pathways for both up and downregulated Genes at 25 and 47 weeks in Myd88 −/− mice. KEGG pathway analysis of up- and downregulated genes in Myd88 −/− mice at 25 (Additional files 3: Figure S2a and b) and 47 weeks (Additional files 3: Figure S2c and d). Significantly enriched pathways (p < 0.05) are presented in each pie graph. (PPTX 1888 kb) [file 12885_2017_3114_MOESM3_ESM.pptx]
